# Primary adrenal insufficiency and myocarditis in COVID-19 disease: a case report

**DOI:** 10.1186/s12902-022-01257-3

**Published:** 2022-12-31

**Authors:** Delaram Eskandari, Amir Ziaee, Abdollah Amirfarhangi Anbardan, Elahe Zeinali, Atefe Tirkan

**Affiliations:** 1 Department of Endocrinology, Rasool Akram Medical Complex, School of Medicine, Iran University of Medical Sciences, Tehran, Iran; 2 Department of Cardiology, Rasool Akram Medical Complex, School of Medicine, Iran University of Medical Sciences, Tehran, Iran; 3 Department of Internal Medicine, Rasool Akram Medical Complex, School of Medicine, Iran University of Medical Sciences, Tehran, Iran

**Keywords:** COVID-19, Primary adrenal insufficiency, Myocarditis, Case report

## Abstract

**Background:**

COVID-19 has different manifestations from respiratory to GI problems, and some of them are more common, but some are rare. Reporting rare cases can significantly advance our understanding of the disease.

**Case presentation:**

In this case, we report an 18-year-old teenage boy with chest pain and resistant hypotension following COVID-19 infection, finally diagnosed as primary adrenal insufficiency and COVID-19 myocarditis.

**Conclusion:**

Adrenal insufficiency can be life-threatening due to its adverse effects on hemodynamic and electrolyte equilibrium. In addition, COVID-19 induced myocarditis can make the situation more complicated.

**Supplementary Information:**

The online version contains supplementary material available at 10.1186/s12902-022-01257-3.

## Background

SARS-CoV-2 (Severe Acute Respiratory Syndrome Coronavirus 2) or COVID-19 (coronavirus disease-19) has endangered millions of lives worldwide and is progressing rapidly day by day. To date, more than 169.5 individuals have been infected with COVID-19 and 3.5 million have died due to its complications [1]. The most common manifestations of the disease involving the respiratory system are cough and shortness of breath, which can progress to respiratory distress and worsen the patient’s status, necessitating mechanical ventilation. In some patients, it can involve other organs such as the heart, GI, owing to the broad distribution of the receptors of SARS-CoV-2 in the body [2, 3].

This case report presents a teenage boy with no past history of adrenal disease or cardiac problems who experienced these two manifestations following COVID-19 infection. Many studies have reported cases of adrenal insufficiency due to COVID-19 severe sepsis, trauma, burns and pancreatitis. Also, some studies have described the association of COVID-19 and different autoimmune conditions such as Guillain-Barre syndrome, immune thrombocytopenic purpura, autoimmune hemolytic anemia, Kawasaki disease, and autoimmune thyroid disease [4]; however, Addison’s disease and the production of antibodies against the adrenal glands after viral infections like COVID-19 is extremely rare, making this case unique. Because of limited sources and knowledge about rare manifestations of COVID-19, its mechanism is still unknown. The uncontrolled production of antibodies against the adrenal glands, which leads to self-destruction of the glands, might be resulted from an imbalance between the immune system and the related suppressor mechanism. In severe cases of COVID-19, the cytokine storm may be the trigger as well. Herein, we report a teenage boy who presented with various common and rare manifestations of COVID-19.

## Case presentation

An 18-year-old boy was admitted to the Rasool Akram Medical Complex on July 20,2020 complaining of shortness of breath, cough, nausea, vomiting, diarrhea and weakness.

As a routine assessment, the SARS-COV-2 RT-PCR (Reverse-Transcription Polymerase Chain Reaction) test was performed which its result was positive. On admission, some other tests were requested to evaluate the patients’ status that their results have been shown in the Table 1. The patient was admitted to the COVID-19 ward and treated with a sofosbuvir and hydroxychloroquine regimen. A few days later, his respiratory status worsened and he was intubated and then treated with steroids and remdesivir. Ten days later, his condition improved, so he was extubated and discharged from the hospital. After 2 weeks, he was referred back to the hospital because of severe weakness, acute chest pain, and ECG abnormalities. His left ventricular ejection fraction (LVEF) was around 30% on echocardiography, accompanied with global hypokinesia. Due to typical acute chest pain and ECG, coronary angiography was performed that found to be normal.

**Table 1:** Lab data on admission

Parameter	Value	Reference range
CRP	> 24	Less than 6 mg/L
ESR	83	Less than 15 mm/h
Ferritin	816	24–336 µg/L
d-Dimer	400	100–250 ng/mL

During the first days of the admission, his blood pressure was 80/40 mmHg not responding to fluid resuscitation. His other symptoms worsened since his first admission. He complained of severe abdominal cramps, ongoing watery diarrhea, headache, and was so weak that he could not to walk unaided.

In the evaluation of his laboratory results, hyponatremia and resistant hyperkalemia were detected (Tables 2 and 3).

**Table 2:** lab data during hospitalization. Note the significant change in electrolyte after treatment

Parameter	Before	Day 1	Day 2	Day 3	Day 4	Reference value
Sodium	129	130	132	140	135	135–145 mmol/L
Potassium	6.2	6.2	6.5	5.4	4.8	3.7–5.5 mmol/L
Creatinine	2.2	1.7	1.7	1.8	1.4	0.5–1.5 mg/dl
WBC	3.1	2.9	2.6	2.9	3.3	4–10 × 10^3^/µL
Hb	8.7	8.3	8.9	9	9	14–18 gr/dl
Platelet	150	167	205	254	213	140–440 × 10^6^/µL

**Table 3:** Hormone levels before starting hydrocortisone and fludrocortisone

Hormone	Value	Reference range
Cortisol 8a.m	1.4	9.3–25 µg/dl
ACTH	> 1800	7.1–56.3 pg/ml
TSH	2.1	0.4–6.1 mIU/L
T4	5.6	4.5–12.5 µg/dl

Moreover, fluid resuscitation, respiratory support, thrombotic prophylaxis, and low dose aspirin (81 mg daily) were considered for him.

More specialized tests were conducted to make a more accurate diagnosis based on his symptoms, laboratory results, and suspicion of adrenal insufficiency. We detected an 8 am serum cortisol level of 1.8 (reference value: 9.3–25 µg/dl) and ACTH level of > 1800 (reference value: 7.1–56.3 pg/ml). The high dose ACTH stimulation test (250 µg intravenous bolus) raised plasma cortisol value from the base level of 1.8 µg/dl to 10.2 µg/dl after 60 min which confirmed the diagnosis of adrenal insufficiency.

**Fig. 1:**
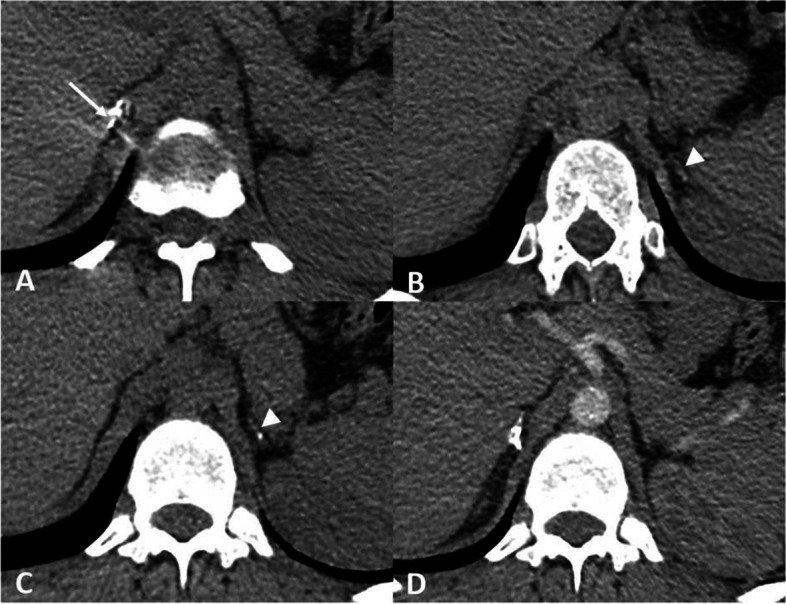
**A**, **B **& **C** abdominal CT scan without contrast (**A**) right adrenal with coarse calcification (**B**) & (**C**) atrophic left adrenal with fine calcification (**D**) abdominal CT scan with contrast (venous phase)

After administering 100 mg of intravenous hydrocortisone, the blood pressure increased, nausea and vomiting and abdominal pain disappeared. Also, the level of electrolytes returned to normal after a few hours. The treatment was continued with 300 mg of intravenous hydrocortisone daily (divided doses). The prescribed amount was reduced to 200 mg daily on the third day. On the fifth day, hydrocortisone was replaced by prednisolone and fludrocortisone (Fig. 1).

We also checked the level of 21-alpha hydroxylase antibodies, which indicated the diagnosis of Addison`s disease. Interestingly, the patient had no evidence of skin or mucosa hyperpigmentation.

The follow-up with echocardiography (eco) after one month showed a significant improvement in LVEF‒ received from 30% in the last echo to 50% in the next echo. The effects of the treatments on decreasing the cardiac chamber size were also significant.

## Discussion and conclusion

Recently published studies have revealed that myocardial damage caused by COVID-19 infection is not rare [5–8]. Zeng et al. reported a 63-year-old male who was admitted with an increased troponin I and general cardiac dyskinesia associated with a decreased LVEF, diagnosed as fulminant viral myocarditis [8]. Elevation of cardiac biomarkers level seems to be associated with a worse prognosis, requiring ICU admission and having a higher risk of sudden deaths in the cases infected with COVID-19 [9, 10].

The mechanism by which viral infections effects on cardiac function is still unknown. However, an elevated systemic inflammatory response and direct infection of the myocardial cells with viral replication can lead to general cardiac stress and isolated cell necrosis in various cardiac regions [11, 12].

In this study, we report a patient with confirmed COVID-19 pneumonia admitted with acute myocarditis and primary adrenal insufficiency.

Based on the studies, different pathogenic microorganisms can damage adrenal structure and function and should be considered as important [13].

Autopsy examination of cases died with SARS-CoV-1 infection revealed that adrenal necrosis with monocytes and lymphocytic infiltration can lead to vasculitis of small veins in the adrenal glands [14, 15]. Additionally, it is speculated that the direct cytopathologic effects of SARS-CoV-2 on adrenal tissue are the result of the high expression of ACE2 (the angiotensin converting enzyme-2) and TMPRSS2 (encoding transmembrane protease serine 2) in the adrenal glands [16, 17]. Hashim et al. reported a case of a 51-year-old male admitted with a history of frequent occurrence of vomiting. On clinical examination, low blood pressure (88/58 mmHg), hyponatremia (sodium 108 mmol/L) and hypochloremia (chloride 78 mmol/L) were detected. Eventually, low morning cortisol level (127 nmol/L) and ACTH stimulation testing confirmed the presence of adrenal insufficiency. Starting a 20 mg daily dose of prednisolone increased the blood pressure and sodium level of the patient [13].

Although clinical features such as pigmentation refer to chronic adrenal insufficiency, lack of it may be in favor of an acute event. However, we agree that there is definite discordance between adrenal imaging and clinical features. In our finding, enlarged adrenal glands with fat stranding in the suprarenal space in COVID-19 related acute adrenal necrosis were shown. This growth can be due to inflammation, a reactive increase in blood flow, and congestion. Small hemorrhagic foci can result in the morphologic changes as well. More specific imaging features of adrenal infarct, such as capsular sign have been described in CT exploration realized at the portal venous phase [18].

It should be considered as important that viral infections can cause multiple organic damages, including cardiac and adrenal injury as our study shown. To the best of our knowledge, this is the first reported case of the adrenal insufficiency with myocarditis following COVID-19 infection.

In conclusion, the high clinical suspicion of the primary adrenal insufficiency should be considered in critically ill COVID-19 patients, especially in those with hypotension unresponsive to fluid therapy, hyponatremia and hyperkalemia. Additional information about the underlying mechanism needs further investigation and brainstorming among all medical centers to make an exact and timely diagnosis for better management of this crucial situation.

## Limitation

While this is an intriguing theory that COVID-19 may be capable of causing Addison’s disease, this case does not establish definitive causation, and early Addison’s disease could also simply have been triggered by severe stress, as shown in this case report. Adrenal atrophy is odd with an acute event, although calcification is nonspecific. As we mentioned earlier, positive adrenal autoantibodies suggest autoimmune adrenalitis. Our case is probably an unmasking of an acute adrenal crisis in the background of subclinical adrenal insufficiency due to an autoimmune process. Delayed onset myocarditis after two weeks of original presentation along with raised inflammatory markers suggests post COVID-19 multisystem inflammatory syndrome (MIS-A) rather than acute myocarditis, but RT-PCR was positive during this presentation [19].

Acute myocarditis due to direct viral cytopathic effects on myocardium. RT-PCR positivity is not odd after 2 weeks, and not against MIS-A [19].

## Supplementary information

**Additional file 1.**

**Additional file 2.**

## Data Availability

The data used to support the findings of this study are included within the article.

## Abbreviations

GI: Gastro Intestinal
SARS-CoV-2: Severe Acute Respiratory Syndrome Coronavirus 2
COVID-19: coronavirus disease-19
LVEF: Left Ventricular Ejection Fraction
ECG: Electro Cardio Gram
MIS-A: Multi System Inflammatory disorder _in adults
ACE2: the angiotensin converting enzyme-2
TMPRSS2: encoding transmembrane protease serine 2
