# Supplementary material for: Primary adrenal insufficiency and myocarditis in COVID-19 disease: a case report
Source: BMC Endocr Disord. 2022 Dec 31;22:336. doi: 10.1186/s12902-022-01257-3 (PMC9805346; doi:10.1186/s12902-022-01257-3)
Supplement: Supplementary file 1 — Additional file 1. [file 12902_2022_1257_MOESM1_ESM.docx]

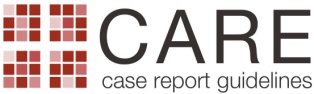
CARE Checklist of information to include when writing acasereport
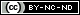


**Topic Item Checklistitem description Reported onLine**

**Title 1** Thediagnosisorinterventionofprimaryfocusfollowedbythewords“casereport” page 1- line 1

**KeyWords 2** 2to5keywordsthatidentifydiagnosesorinterventionsinthiscasereport,including"casereport" page 2–line34

# Abstract

**(no references)**

**3a** Introduction:Whatisuniqueaboutthiscaseandwhatdoesitaddtothescientificliterature? Page3–line57-63&133-7

**3b** Mainsymptomsand/orimportantclinicalfindings page4–line72-80

**3c** Themaindiagnoses,therapeuticinterventions,andoutcomes page4&5–line89-104

**3d** Conclusion—Whatisthemain“take-away”lesson(s)fromthiscase?……………………………………………..page6–line133-37

**Introduction 4** Oneortwoparagraphssummarizingwhythiscaseisunique(**mayincludereferences**) page3–line57-63

**PatientInformation 5a** De-identifiedpatientspecificinformation N/A

**5b** Primary concerns and symptoms of thepatient page4 -line72-80

**5c** Medical,family,andpsycho-socialhistoryincludingrelevantgeneticinformation page 4–line71-76

**5d** Relevant past interventions withoutcomes page4 -line71-76

# Clinical Findings

**Timeline**

**Diagnostic Assessment**

**Therapeutic Intervention**

**Follow-up and Outcomes**

1. Describe significant physical examination (PE) and important clinicalfindings …………….page4-line71-73and page5-line101-102
2. Historicalandcurrentinformationfromthisepisodeofcareorganizedasatimeline page 3–line49-55&page6-line 125-130

**8a** Diagnostic testing (such as PE, laboratory testing, imaging,surveys). page11& page 12

**8b** Diagnostic challenges (such as access to testing, financial, orcultural) …………………………..page4–line92-94

**8c** Diagnosis (including other diagnosesconsidered) page 4–line 92-94

**8d** Prognosis (such as staging in oncology) whereapplicable N/A

**9a** Typesoftherapeuticintervention(suchaspharmacologic,surgical,preventive,self-care) page5–line95-99

**9b** Administrationoftherapeuticintervention(suchasdosage,strength,duration) page5–line95-99

**9c** Changesintherapeuticintervention(withrationale)page4-line 87-88&page5(line95-99)

**10a** Clinicianandpatient-assessedoutcomes(ifavailable) N/A

**10b** Importantfollow-updiagnosticandothertestresults page4–line92-94

**10c** Interventionadherenceandtolerability(Howwasthisassessed?) N/A

**10d** Adverseandunanticipatedevents N/A

**Discussion 11a** AscientificdiscussionofthestrengthsANDlimitationsassociatedwiththiscasereport….page5&6–line107-137andpage 7-line140-142

**11b** Discussionoftherelevantmedicalliterature**withreferences** page5&6–line-107-137

**11c** Thescientificrationaleforanyconclusions(includingassessmentofpossiblecauses) page6 -line134-138

**11d** Theprimary“take-away”lessonsofthiscasereport(withoutreferences)inaoneparagraphconclusion page6–line134-138

**PatientPerspective 12** Thepatientshouldsharetheirperspectiveinonetotwoparagraphsonthetreatment(s)theyreceived N/A

**InformedConsent 13** Didthepatientgiveinformedconsent?Pleaseprovideifrequested...................................... **Yes * No**
