# Supplementary figures and images for: Primary adrenal insufficiency and myocarditis in COVID-19 disease: a case report
Source: BMC Endocr Disord. 2022 Dec 31;22:336. doi: 10.1186/s12902-022-01257-3 (PMC9805346; doi:10.1186/s12902-022-01257-3)

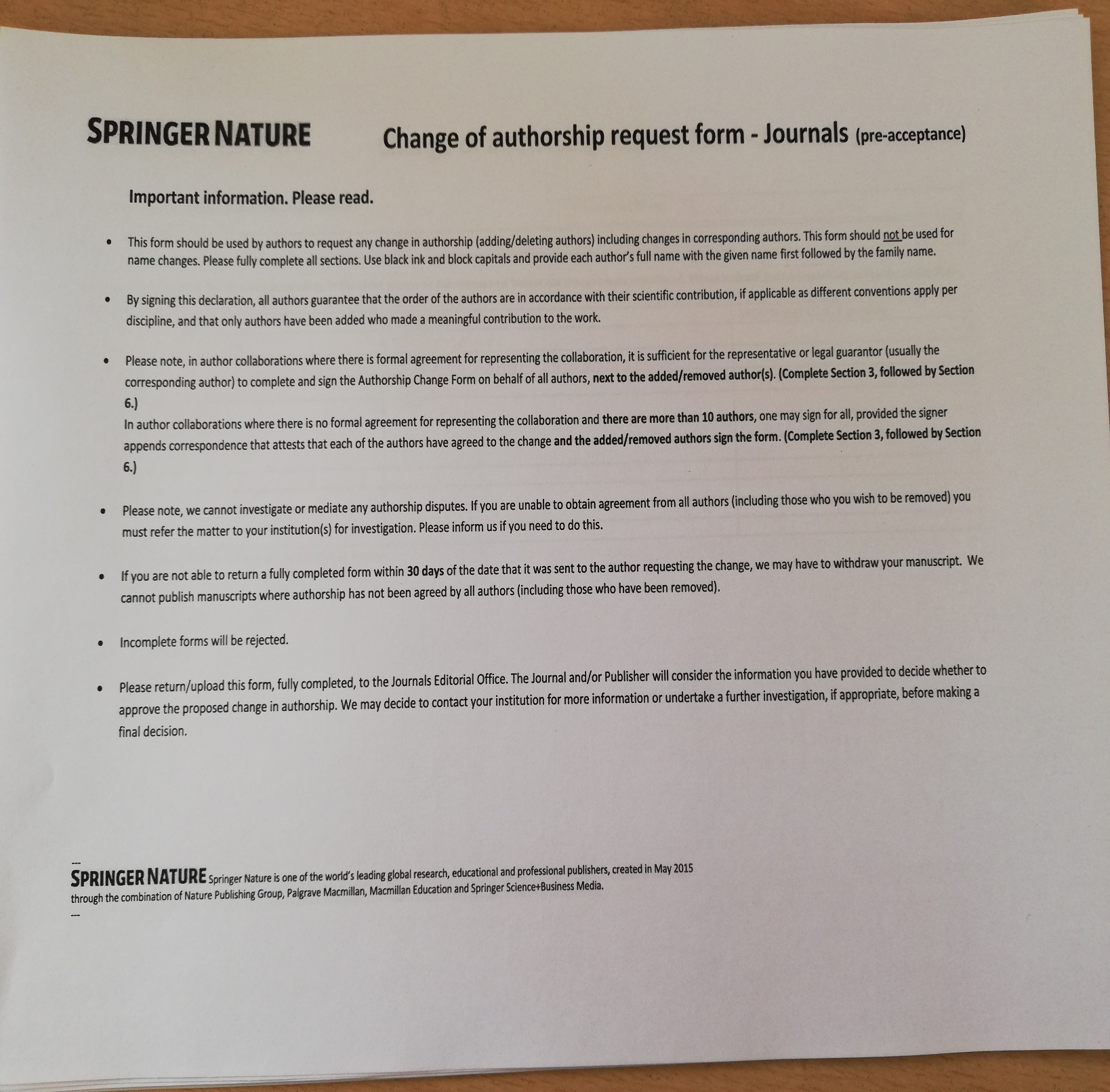


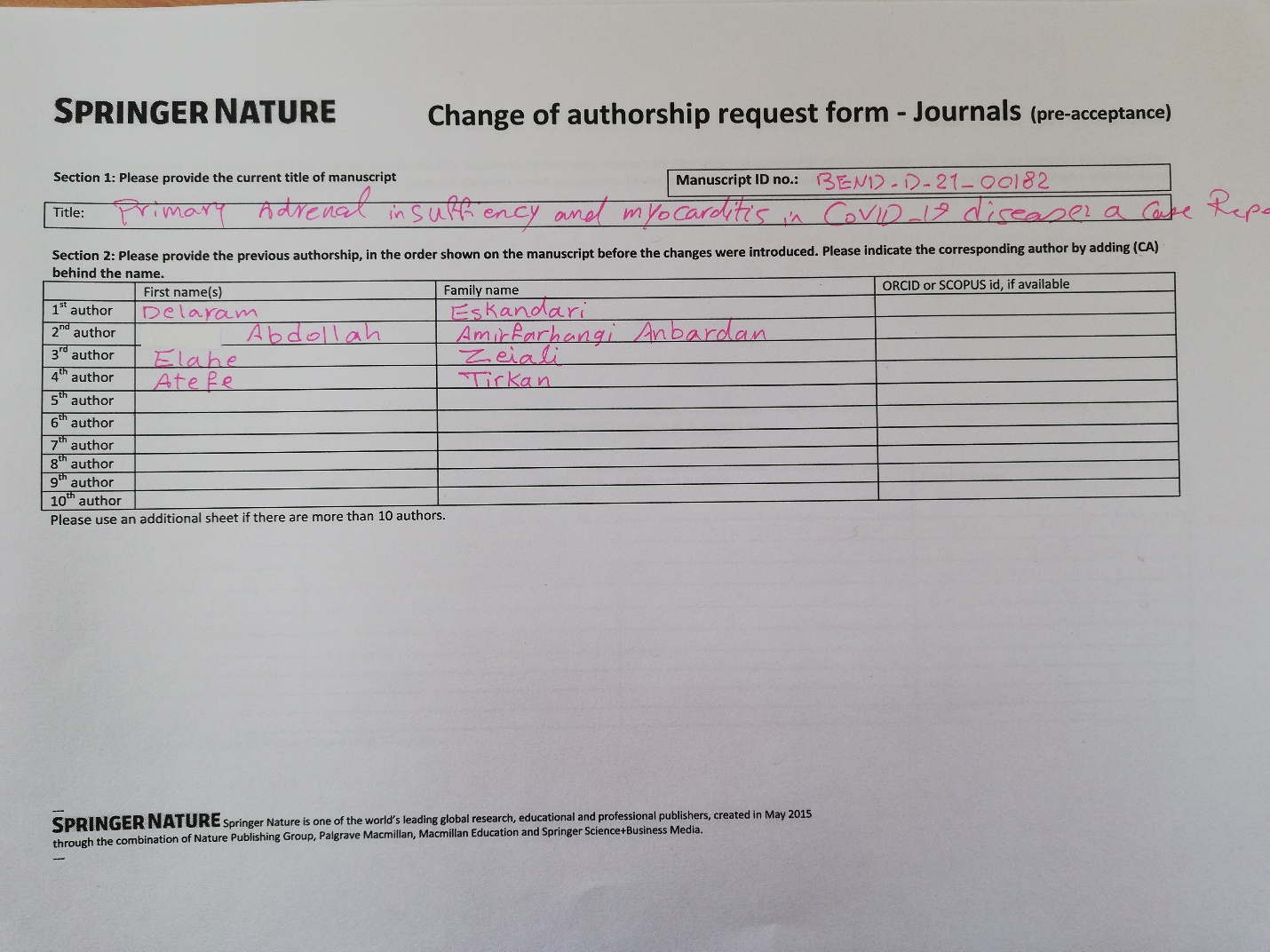


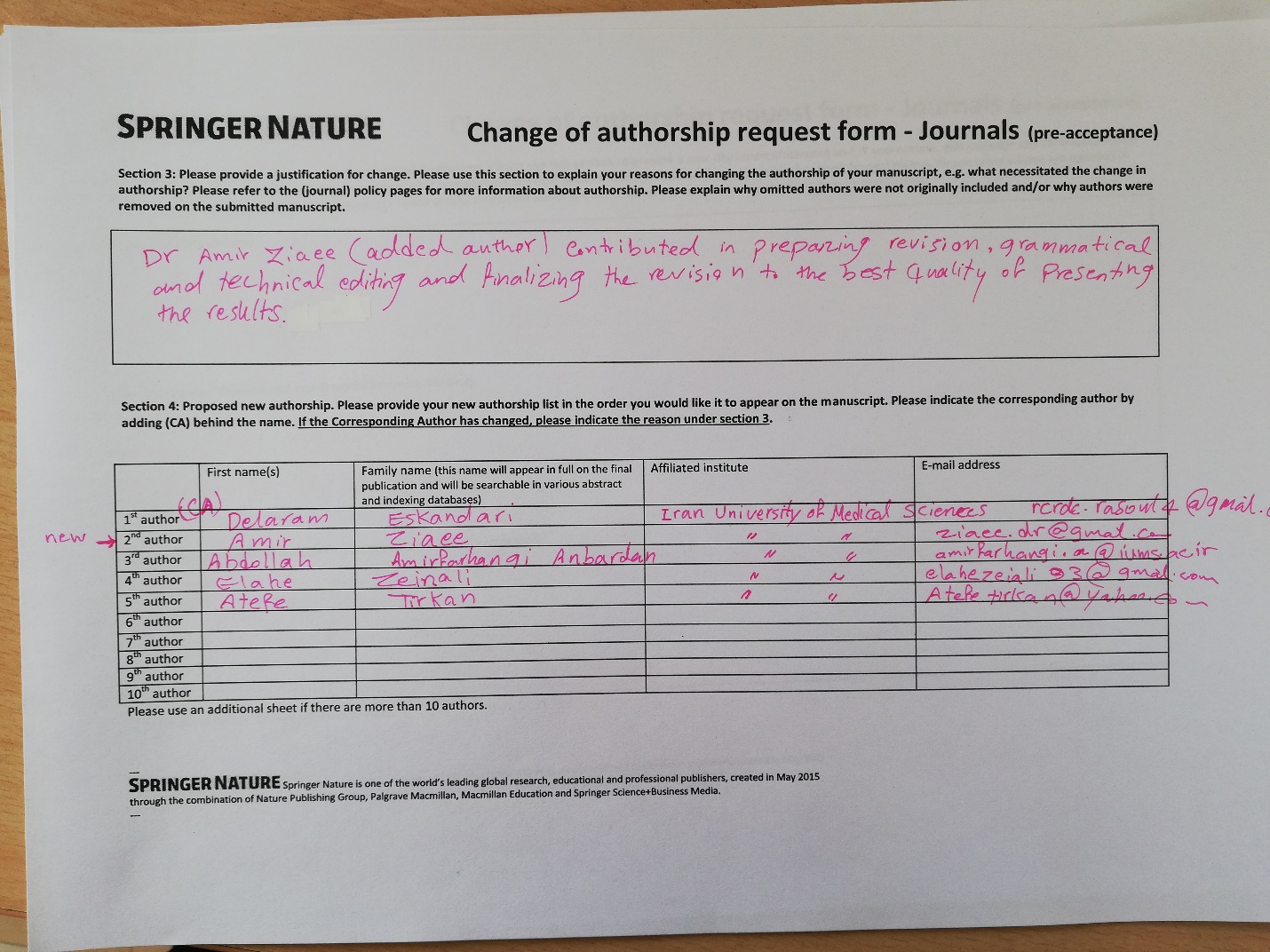


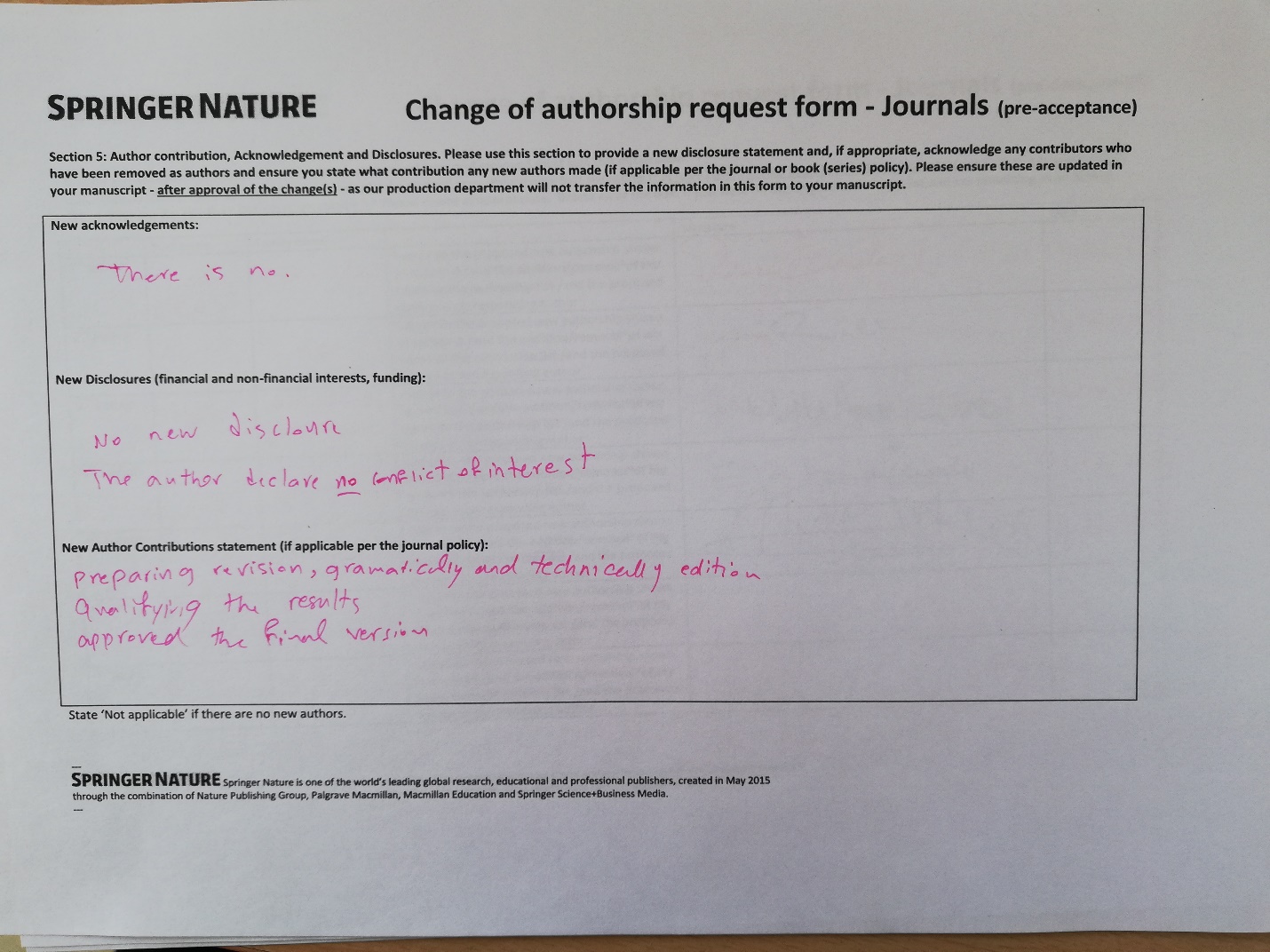


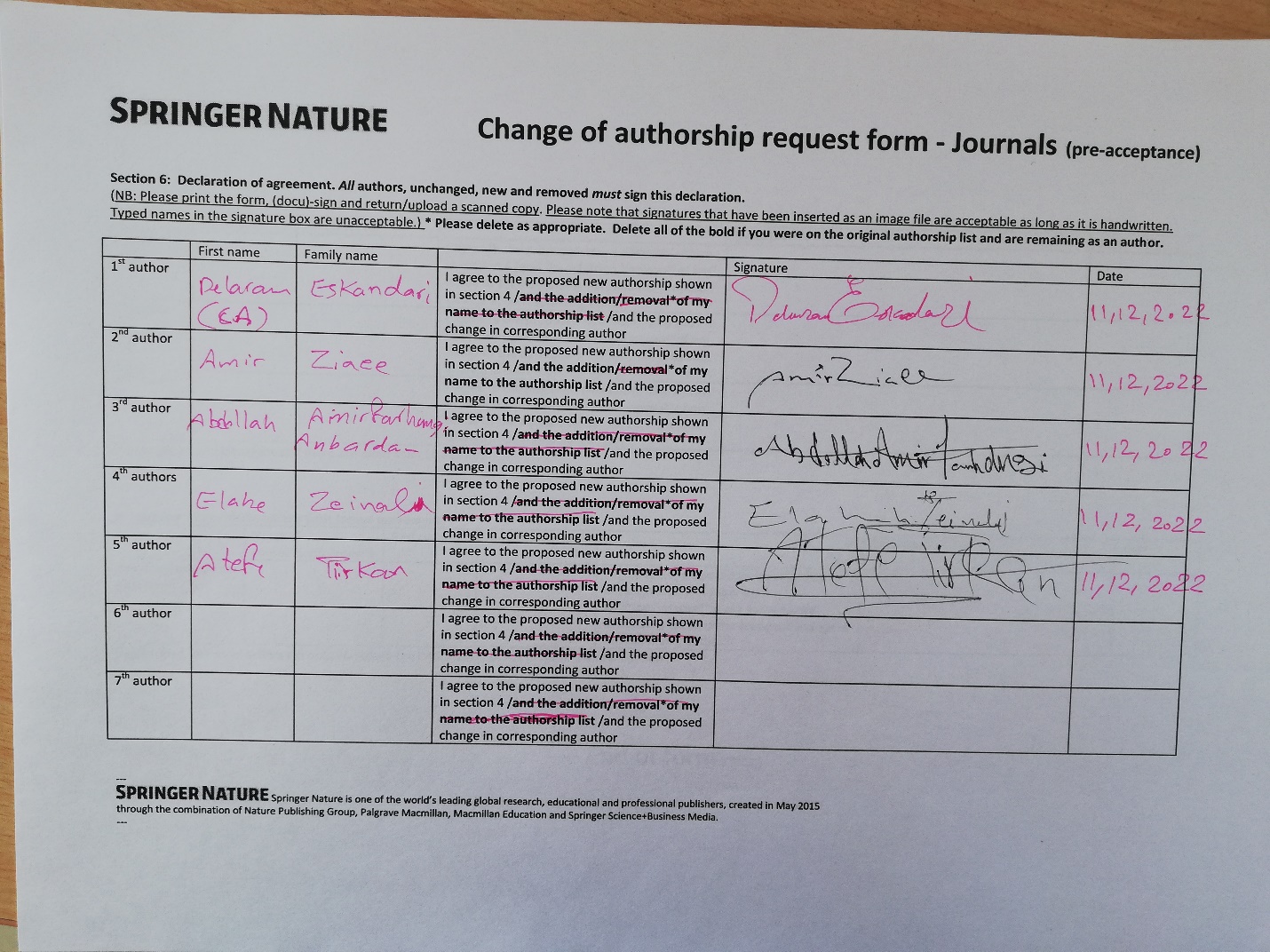


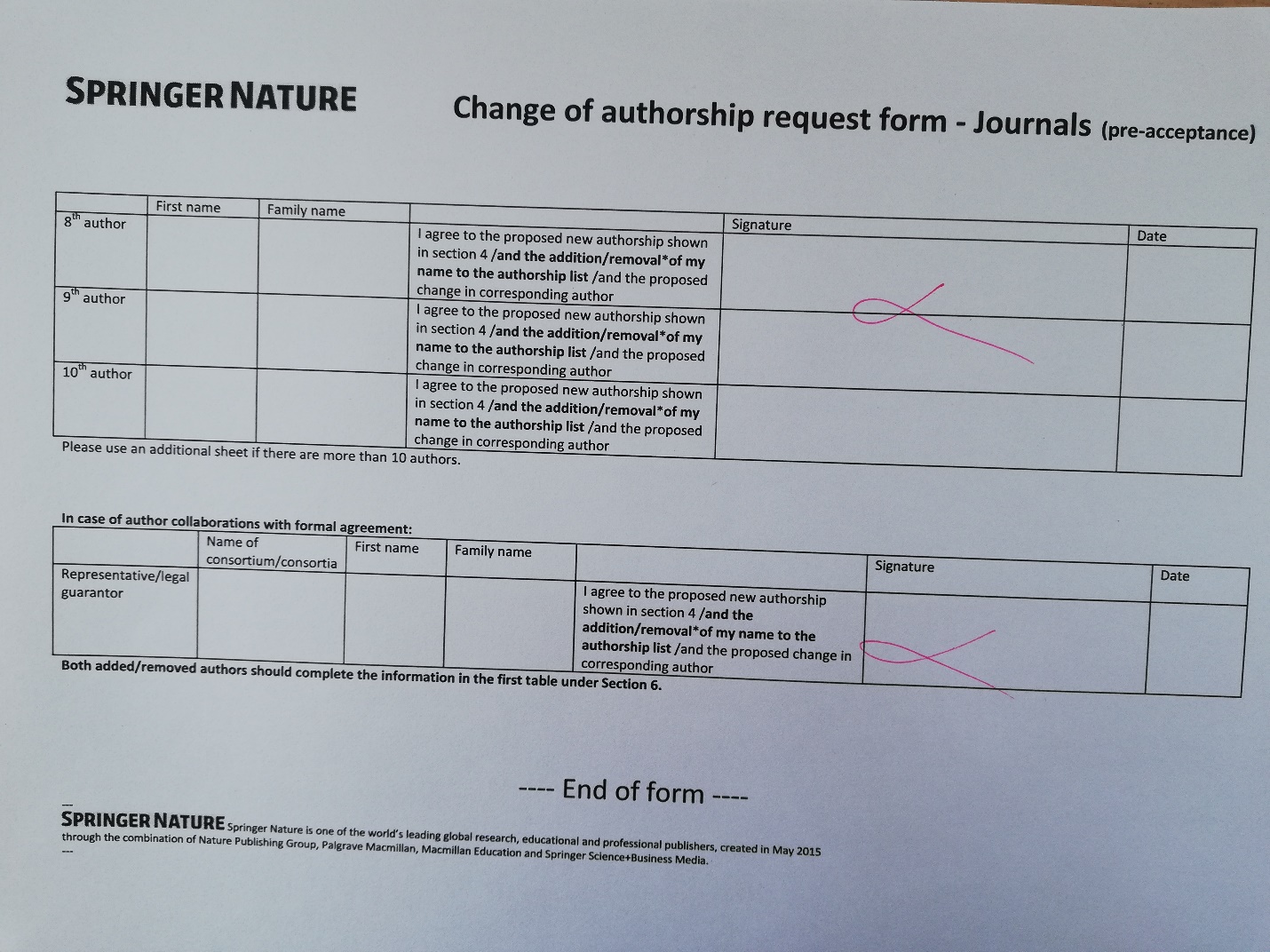

Supplement: Supplementary file 2 — Additional file 2. [file 12902_2022_1257_MOESM2_ESM.docx]
